# Comparative Efficacy of Cultivated Epidermal Allograft and Hydrocolloid Dressings in Pediatric Burn Injuries

**DOI:** 10.7759/cureus.102892

**Published:** 2026-02-03

**Authors:** Hugo E Beyuma-Mora, Héctor E Arriaga C, Maria A Chavez-Suarez, Miguel de la Parra-Marquez, Myrna L Cuevas, Héctor Ramsés López-Lazcano, Diego Montemayor, Sergio Charles-Lozoya

**Affiliations:** 1 Plastic and Reconstructive Surgery, Instituto Mexicano del Seguro Social, Unidad Médica de Alta Especialidad, Hospital de Traumatología y Ortopedia No. 21, Monterrey, MEX; 2 Vicerrectoría de Ciencias de la Salud, Universidad de Monterrey, San Pedro Garza García, MEX; 3 Research, Instituto Mexicano del Seguro Social, Unidad Médica de Alta Especialidad, Hospital de Traumatología y Ortopedia No. 21, Monterrey, MEX; 4 Vicerrectoría de Ciencias de la Salud, Universidad de Monterrey, San Pedro Garza Garcia, MEX

**Keywords:** burn child, burn wound, hydrocolloid dressing, pediatric patients, surgical wound dressing

## Abstract

Introduction: Managing burns in pediatric patients is crucial due to their unique physiology and higher risk of complications compared to adults. Key components of burn management for pediatric patients involve assessing the extent of the burn injury, administering appropriate pain management, and closely monitoring for signs of infection. Comprehensive burn management includes securing an adequate airway, administering appropriate anesthesia, performing surgical debridement, repairing damaged regions with skin grafts, providing nutritional support, and facilitating rehabilitation for optimal recovery. Dressing-based therapy is essential in comprehensive burn care, as it promotes wound healing, prevents infection, reduces pain during dressing changes, and maintains a moist wound environment for optimal recovery. Ideal dressings should promote wound healing, possess antibacterial properties, minimize discomfort during changes, and effectively alleviate pain. A study was conducted to evaluate the effectiveness of treating pediatric burn wounds using cultivated epidermal allografts compared to hydrocolloid dressings, aiming to assess the necessity for skin grafting and identify potential complications.

Methods: A retrospective cross-sectional study was conducted on pediatric patients with burn injuries. The analyzed variables included age, sex, burn mechanism, percentage of total body surface area (TBSA) affected, anatomical site, burn depth, length of hospital stay, type of dressing used, time to complete epithelialization, need for skin grafting, admission to the intensive care unit (ICU), and mortality. Patients were categorized into two groups: those treated with cultivated epidermal allografts and those treated with hydrocolloid dressings. Inferential statistical analyses were performed to assess the association between the type of treatment and the necessity for skin grafting.

Results: A total of 65 patients were included. The most common age group was preschoolers (1-5 years) , with a median age of three years, representing 64% of cases. Males were more affected. Scalds were the most frequent burn mechanism (64%; n=42). The median TBSA affected was 8%, and second-degree burns (both superficial and deep) were the most prevalent (55%). The trunk was the most affected area. In the cultivated epidermal allograft group, the median time to epithelialization was eight days, and the median hospital stay was seven days. In contrast, the hydrocolloid group had a median epithelialization time of 18 days and a median hospital stay of 10.5 days. Only 19% (n=4) of patients in the cultivated epidermal allograft group required skin grafting, compared to 50% (n=11) in the hydrocolloid group (p=0.016).

Conclusion: Cultivated epidermal allograft showed a tendency towards being beneficial for managing partial-thickness and indeterminate-depth burns, promoting faster epithelialization, preventing burn progression, and reducing the need for skin grafts. Its use was also associated with shorter hospital stays and potentially lower healthcare costs.

## Introduction

Data from the Global Burn Registry indicates that nearly half of all individuals affected by burns worldwide are under the age of 18. Among this population, children between the ages of one and five are the most vulnerable, accounting for 62% of pediatric burn cases. Furthermore, approximately 52% of the children included in the report sustained severe burns involving at least 15% of their total body surface area (TBSA) [1]. Due to their early developmental stage, marked by increased mobility, curiosity, and their limited understanding of danger, children have a high incidence of burn injuries. Common mechanisms, often occurring in the home, include contact with hot surfaces, direct flame exposure, and scalds from hot liquids and beverages [2]. In low- and middle-income countries, where healthcare systems frequently lack the resources to provide specialist care, burn injuries continue to be a significant global public health concern. Burns have a major socioeconomic impact on families and healthcare systems, making them one of the primary causes of morbidity and extended hospitalization in the pediatric population [3].

Depending on the severity of the injury, the host's initial response to a major burn resembles and generally exceeds the response observed in other inflammatory conditions triggered by tissue destruction [4], such as trauma or major surgery. This early inflammatory response is beneficial for initiating tissue repair and wound healing [5]. However, if this inflammatory cascade is activated repeatedly or remains dysregulated, it may result in host tissue damage, organ dysfunction, and even death. While several components of the complex burn response have been identified, their precise interactions and chronological sequence remain incompletely understood [6].

The management of burns in pediatric patients requires special attention due to the unique physiological characteristics of this population. Pediatric skin is thinner than adults', predisposing children to deeper and more extensive burns, as well as higher morbidity due to scar contractures [7].

Comprehensive burn treatment involves appropriate airway management, sedation, surgical debridement, reconstruction of affected areas, nutritional support, and rehabilitation [8]. Surgical management specifically includes early debridement and timely wound coverage using suitable dressings, skin grafts, or flaps [9].

Dressings are protective or therapeutic materials applied to a wound that may mimic components of the skin and its functions [10]. The primary goals of dressings are to absorb fluid, regulate moisture around the wound, serve as antibacterial barriers, and provide mechanical protection from outside pollutants, thereby limiting infection and inflammation while alleviating pain [11].

Throughout their existence, burn dressings have progressed from being straightforward coverings to being made of complex materials that are intended to promote healing and minimize problems. The early methods of treating burns consisted of using simple cloth or gauze to shield wounds; however, these methods offered only limited relief from pain and infection. As time went on, advancements led to the introduction of dressings that possessed antibacterial characteristics and retained moisture [12].

Wound exudate is crucial when choosing a dressing. Hydrocolloids and hydrogels are most effective for low to moderate exudate. For partial-thickness burns with moderate to high exudate, foams and alginates are advised. Moderate- to high-exudate wounds, particularly recent burns that received insufficient or no first aid, can benefit from the use of iodine or silver dressings [13].

Globally, the most valued characteristics of an ideal burn dressing include antimicrobial capacity, pain-free dressing changes, and pain reduction. However, there are regional differences in the perception of these priorities. In Africa and Asia, the dressing's non-adherence to the wound was considered one of the three most important features. In Africa, antimicrobial capacity was ranked as less critical compared to other regions and the global control group [14].

Hydrocolloid dressings are made up of a mix of colloidal substances, elastomers, and alginates. These dressings are typically biodegradable and biocompatible, making them suitable for the treatment of superficial wounds such as minor burns, blunt trauma injuries, and contusions. They are occlusive, preventing the entry of water, bacteria, and oxygen into the wound. Additionally, they contribute to lowering the pH of the affected area, which may inhibit bacterial growth. These dressings are easy to apply and adhere well to the skin [15].

A 2023 study looked at how well hydrocolloid dressings worked compared to silver sulfadiazine in children with scald burns. The average age of participants was 2.88 years, and most were treated on an outpatient basis. Results indicated that hydrocolloid dressings were more effective: they promoted complete healing in less time, reduced the number of dressings required, and decreased the need for partial-thickness skin grafts after three weeks [16].

Premanufactured cultured epidermal allografts, derived from in vitro expanded human skin cells, represent an advanced therapeutic option for burn wound coverage. These allografts consist of sheets of cultured human keratinocytes grown under controlled laboratory conditions. Their mechanism of action is similar to that of conventional cryopreserved human epidermal allografts and primarily relies on the release of growth factors that promote wound healing [17].

Significant advances have been made in human keratinocyte culture techniques, including both two-dimensional monolayers and three-dimensional organotypic models. Monolayer cultures enhance keratinocyte proliferation, whereas organotypic models more closely replicate native tissue environments, supporting cell differentiation and the development of stratified cellular layers [18].

Evidence suggests that biosynthetic dressings may be more effective than silver sulfadiazine cream in promoting healing and alleviating pain; however, this evidence is derived from small, low-quality studies. There is insufficient data to determine whether biosynthetic dressings are superior to hydrocolloid dressings for wound healing or pain control [19].

In pediatric patients, dressing-based treatment is a key component of comprehensive burn care [20]. Among the available options, hydrocolloid dressings and cultivated epidermal allografts have shown particular efficacy in the management of pediatric burns [21].

The study aimed to compare the results of treating burn wounds with the cultivated epidermal allograft and other types of dressings in pediatric patients.

## Materials and methods

This observational retrospective study was conducted at Hospital de Traumatología y Ortopedia No. 21, in Monterrey, Mexico.

Study population

**Figure 1:**
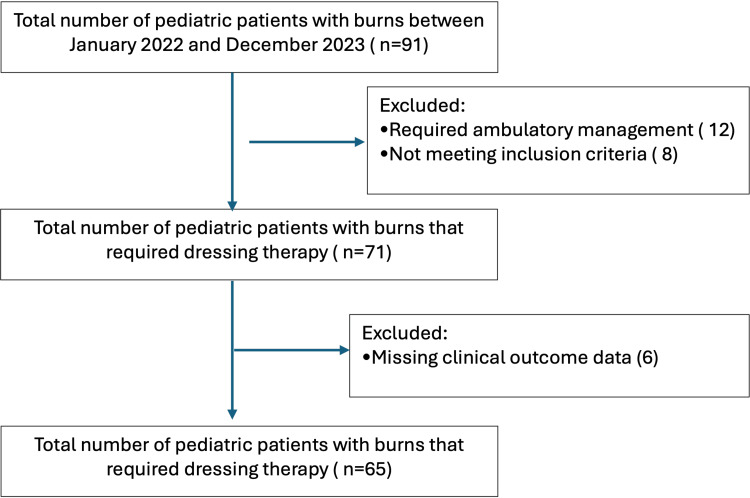
STROBE flowchart STROBE: Strengthening the Reporting of Observational Studies in Epidemiology

The study included medical records of patients under the age of 18 who were hospitalized for burn treatment between January 2022 and December 2023 (Figure 1). Patients were admitted for management of burn injuries based on institutional clinical criteria. These included extensive burns involving more than 10% TBSA in pediatric patients, deep electrical burns with exposure to underlying tissues, chemical burns, burns in special anatomical locations (such as the face, genitalia, or joints), and patients from remote areas requiring inpatient care. Exclusion criteria included full-thickness burns requiring immediate surgical management, burns with exposed underlying structures (e.g., tendon, muscle, or bone) at presentation, documented wound infection at initial assessment, and incomplete or inconsistent medical records, including missing primary outcome data (time to epithelialization or need for grafting).

Procedures

In the absence of clinical signs of infection, such as discoloration of the wound, progression to a deeper burn, exudate or purulence, local warmth, swelling, or fever, wound dressing was applied to all second-degree burns (both superficial partial-thickness and deep partial-thickness) within 48-72 hours post-injury. Patients were assigned to hydrocolloid or cultivated epidermal allograft dressings based solely on hospital availability at the time of treatment, disregarding patient characteristics or clinical severity, thus minimizing the risk of selection bias. The attending plastic and reconstructive surgeon in the emergency department determined the treatment modality. Epithelialization was deemed complete at the discretion of attending physicians when the wound bed, which had previously been moist, reddened, and tender, transitioned to a dry, shiny, hypopigmented epithelium that exhibited neither tenderness nor bleeding, within a maximum healing period of 21 days. The attending physician monitored the patients every three weeks following discharge.

Application of cultivated epidermal allograft

Cultivated epidermal allograft is a biological dressing composed of human keratinocyte sheets obtained from cryopreserved cell banks originally derived from neonatal foreskin tissue [22]. The selected keratinocytes are cultured in vitro under controlled conditions for approximately 12 days, generating multilayered viable cell sheets mounted on a sterilized, Vaseline-impregnated gauze, protected between two sterile polyethylene plastic grills, forming the physical structure of the patch. Each batch undergoes strict quality control to verify hermetic sealing, sterility, and epithelial integrity according to standardized protocols. The patches are packaged in double sterile polyethylene bags and frozen at -70°C±10°C, with a shelf life of up to 24 months at -70°C or 12 months if stored between -20°C and -60°C [23]. 

Wound Preparation

The wound was cleaned with a sterile saline solution and povidone-iodine antiseptic, and gentle debridement was done in the operating room under sterile conditions to reveal the partial-thickness burn, making sure the wound area was clean and not infected as noted by the attending staff. There had been no previous treatment of the burn injury.

Dressing Preparation

Epifast® (cultivated epidermal allograft) by Bioskinco (Guadalajara, Mexico) was prepared for application according to the manufacturer's instructions [24].

Application

The dressing was cut to fit the size of the wound (if needed), with a 1-2 cm overlap onto healthy skin, and then placed directly over the lesion. Once applied, it cannot be moved or taken off without losing its bioactive properties.

Fixation

The dressing was covered with sterile non-adherent secondary dressings (sterile paraffin-impregnated gauze) and secured with a soft bandage without applying excessive pressure. Splinting was sometimes necessary, especially in burns in flexion areas or areas with a high risk of dressing displacement.

Post-application Care

A daily visual inspection of the bandages covering the dressings was conducted. Bandages were removed in the presence of significant exudate or a fetid odor. With complete epithelialization, the patches exhibit dryness and can be removed with ease. In the absence of complete epithelialization, the prior patches were removed, the burn was treated aseptically, and new patches and bandages were applied for an additional five days.

Application of hydrocolloid dressing (DuoDERM®, ConvaTec Inc., London, United Kingdom) [25]

Wound Preparation

The wound was cleansed with sterile saline and povidone-iodine, followed by gentle debridement using sterile gauze and compresses. Surgical debridement (cutting and sharp dissection) was then performed in the operating room under sterile conditions to remove burned skin and expose the partial-thickness burn. The wound bed was confirmed to be clean and free of active infection. No prior manipulation of the burn had been performed.

Dressing Selection and Trimming

A hydrocolloid dressing of appropriate size was selected and trimmed to ensure at least a 1-2 cm overlap onto healthy skin to promote effective adhesion.

Application

The protective backing of the dressing was removed, and the dressing was applied directly over the wound without stretching the material. Care was taken to avoid air bubbles and to ensure that the dressing conformed to the contours of the skin.

Fixation

This hydrocolloid dressing is self-adhesive; additional fixation was not typically required. However, due to profuse burn exudate, especially in areas subject to friction or movement, a secondary absorbable dressing and a light bandage (cotton gauze and pads as a secondary dressing) were used to reinforce the dressing. Splinting was sometimes necessary, especially in burns in flexion areas or areas with a high risk of dressing displacement.

Post-application Care

The dressing was left in place for 5-7 days, depending on the level of exudate. Removal was performed gently by lifting the edges and pulling parallel to the skin to minimize trauma.

Variables

The following variables were collected from patient records: age, sex, burn mechanism, percentage of TBSA burned, anatomical burn site, burn type, hospital stay length (in days), dressing type used, days to complete epithelialization, need for skin grafting, admission to the intensive care unit (ICU), and mortality. Among these, the need for skin grafting, length of hospital stay, ICU admission, and mortality were analyzed as the main clinical outcomes and complications documented in patient records.

Data collection

Data were manually extracted using a standardized collection form designed for this study. All patient information was anonymized and handled confidentially in accordance with institutional guidelines. While data extractors were not blinded to treatment assignments, they remained unaware of the study's primary outcomes or preliminary analyses.

Statistical analysis

Categorical variables were summarized using frequencies and percentages. Numerical variables were described using measures of central tendency (mean or median) and dispersion (standard deviation or interquartile range), according to their distribution. Normality of numerical variables was assessed through the Shapiro-Wilk test.

Bivariate analyses were performed to explore associations between variables. The chi-squared test or Fisher's exact test was applied for categorical variables, and Student's t-test or the Wilcoxon rank-sum test was used for numerical variables. A p-value of <0.05 was considered statistically significant.

All statistical analyses were performed using IBM SPSS Statistics for Windows, Version 23.0 (IBM Corp., Armonk, New York, United States).

Ethical considerations

This study was approved by the Mexican Social Security Institute (approval number: R-2025-1903-021). It was conducted in accordance with the principles of the Declaration of Helsinki. Given its retrospective nature, informed consent was not required.

## Results

There were 65 patients included in the study. The median age of the patients was three years (IQR: 1.5-6.5), with a predominance of males, accounting for 40 (61.5%) patients. The most common burn mechanism was scalding, observed in 42 (64.6%) cases, and the most frequent site of burns was the trunk in 38 (58.5%). The hydrocolloid dressing was used on 44 (67%) of the patients, making it the most common type of dressing. The median percentage of TBSA burned was 8% (IQR: 4.5-15.9). A total of 15 (23%) patients required admission to the ICU. The median length of hospital stay was nine days (IQR: 5-19.5). The median time to complete epithelialization was 16 days (IQR: 8-24.5). A total of 26 (40%) patients required skin grafting. No deaths were reported during the study period. For all the descriptive statistics, see Table 1.

**Table 1:** General characteristics of the 65 pediatric patients admitted for the management of burn injury in the Hospital de Traumatología y Ortopedia No. 21 TBSA: total body surface area; ICU: intensive care unit

Patients' general characteristics		
Sex, male (n, %)		40 (61.5)
Age (median, IQR)		3 (1.5-6.5)
Age ranges	Less than 1 y/o	4 (6.2)
	1-5 y/o	42 (64.6)
	6-12 y/o	14 (21.5)
	13-18 y/0	5 (7.7)
Burn mechanism	Scalding	42 (64.6)
	Flame	11 (16.9)
	Electrical	3 (4.6)
	Contact	6 (9.2)
	Friction	3 (4.6)
TBSA (median, IQR)		8% (4.5-15.5)
Site of burn	Face	30 (46)
	Neck	13 (20)
	Trunk	38 (58.5)
	Upper limb	29 (44.6)
	Hand	18 (27.7)
	Lower limb	25 (38.5)
	Genitalia	8 (12.3)
	Perineum	6 (9.2)
Burn degree	2nd	48 (73.8)
	3rd	17 (26.2)
Skin graft (n, %)		26 (40)
ICU admission (n, %)		15 (23.1)

Patients who received cultivated epidermal allograft dressings exhibited a markedly reduced necessity for skin grafting in comparison to those treated with hydrocolloid dressings (19% vs. 50%; χ²=5.84; p=0.016). Patients in the cultivated epidermal allograft group exhibited a shorter median hospital stay (7 days vs. 10.5 days; Z=-0.46; p=0.65) and a reduced duration to achieve complete epithelialization (8 days vs. 18 days; Z=-0.97; p=0.33), although these differences were not statistically significant. Additional comparisons are presented in Table 2.

**Table 2:** Comparison of the use of cultivated epidermal allograft vs. hydrocolloid dressing in 65 pediatric patients admitted for the management of burn injury in the Hospital de Traumatología y Ortopedia No. 21 ICU: intensive care unit

	Cultivated epidermal allograft (21)	Hydrocolloid (44)	P-value
Skin graft (n, %)	4 (19)	22 (50)	0.016
Epithelization days (mean, IQR)	8 (5-30)	18 (4-60)	0.33
Hospitalization days (mean, IQR)	7 (1-21)	10.5 (1-43)	0.65
ICU admission (n, %)	4 (19)	11 (25)	0.42

## Discussion

The cultivated epidermal allograft showed a trend toward improved outcomes, including a reduced need for skin grafting, shorter length of stay, and fewer days for epithelialization compared to hydrocolloid dressings. These results indicate that cultivated epidermal allografts may offer considerable advantages in wound management, potentially resulting in improved patient recovery experiences and more effective use of healthcare resources. Male children are reported to be more likely to experience these effects than female children [26]. A Mexican article indicates that scalding remains the most common cause of burns in this population [27]. Patients treated with cultivated epidermal allografts had a lower likelihood of requiring a skin graft compared to those who received hydrocolloid dressings. This parallels findings from a study that demonstrated significantly superior healing rates, percentage reductions in initial ulcer size, and radial progression toward wound closure for cultivated epidermal allografts versus hydrocolloids in patients with venous ulcers [28]. Furthermore, cultivated epidermal allografts appear to improve epithelialization time relative to other studies, which report means or medians of around 9-14 days for over 90% wound closure [29,30]. Few studies compare different dressing protocols, such as bismuth tribromophenate, and their association with ICU admissions [31]. Nevertheless, our observations indicate favorable outcomes with cultivated epidermal allografts in preventing ICU admissions when compared to hydrocolloid dressings.

The average epithelialization time for patients treated with cultivated epidermal allografts was eight days (±7.2), which aligns closely with the findings of Rodriguez-Ferreyra et al.'s study in a Mexican population [32], where the reported time was 7.5 days (±4.2).

Gauze dressings are typically constructed from loosely woven cotton fibers, making them highly absorbent, breathable, and flexible. They are frequently utilized as either primary or secondary wound dressings and can be modified to enhance their characteristics. Cotton dressings encompass not only gauze but also absorbent cotton pads and non-woven cotton, which can vary in density, absorbency, and intended applications [33]. While traditional gauze dressings primarily serve to protect wounds from external damage without actively promoting healing, modern dressings are designed to create a moist environment that aids recovery and reduces the risk of bacterial infection. This evolution has significantly advanced wound care practices. Today, a diverse selection of dressings is available, including films, foams, hydrogels, hydrocolloids, alginates, and bioactive options [34]. Choosing an appropriate dressing requires a thorough assessment of the wound's characteristics, including its type, depth, and exudate production, the presence of infection, and the overall health of the patient [35]. 

Hydrocolloid dressings, made from colloidal materials with elastomers and alginates, are usually biodegradable and biocompatible, suitable for superficial wounds such as minor burns, trauma injuries, and bruises, as they absorb exudate and maintain a moist environment [36]. Skin equivalents can aid wound healing [37], promote cellular migration, and release growth factors, as they are composed of cultured keratinocytes that form an epidermal layer without dermal components, thereby encouraging cell proliferation from ulcerated skin to facilitate re-epithelialization [38].

Pediatric burn injuries are a significant public health concern, particularly for children under five years of age, who represent the most vulnerable subgroup. Scald injuries are the most common cause of burns in this age group. The demographic characteristics of our study cohort align with the findings of Garcia Garcia et al. [39], who performed a retrospective cohort study of pediatric burn patients admitted to public hospitals in Mexico.

Children's skin is thinner than adults', making them more susceptible to burns that occur more easily and penetrate more deeply. For example, a liquid at 140°F (60°C) can cause a third-degree burn in just three seconds [40]. In a retrospective review of patients seen at a level II pediatric trauma center between January 1, 2017, and December 31, 2019, second-degree burns accounted for 79% of all burn injuries [41]. In our study, the distribution of burn severity was similar, with second-degree burns also being the most common.

In a study of inpatients from 196 hospitals in China (2009 and 2018), the most frequently affected burn site was the upper limb. In contrast, our study identified the trunk as the most affected anatomical region, followed by the upper limb, including the hand. However, we consider this difference to be relatively minor and likely attributable to variations in study populations or injury circumstances [42].

**Figure 2:**
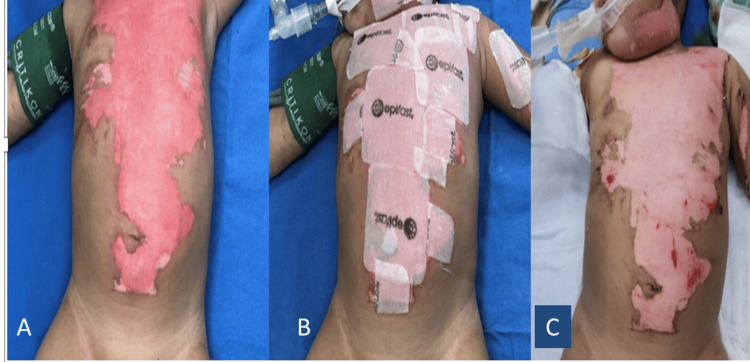
(A) Partial-thickness burns involving the trunk, neck, face, and upper extremity. (B) Surgical debridement and application of cultivated epidermal allograft (Epifast®). (C) Complete epithelialization eight days after treatment with cultivated epidermal allograft

Skin grafts play a critical role in burn wound management by reducing infection risk, minimizing scarring, and improving both function and aesthetic outcomes (Figure 2). In pediatric patients with deep or extensive burns, skin grafting is often necessary to promote healing and prevent complications [43]. Deep burns that do not heal within 21 days almost invariably require grafting to facilitate wound closure, shorten hospital stays, reduce infection risk, and improve functional recovery and scar quality [44].

Limitations of the study

We must view these findings with caution due to various constraints that may impair their generalizability. The primary limitation of the study is its observational design, which inhibits the identification of causal relationships among the variables. The data were sourced from a population concentrated in a specific region of Mexico, potentially lacking representation of larger or more diverse populations. A further significant factor is the limited sample size. Due to the small number of comparisons, the analysis was exploratory, and no adjustments for multiple comparisons (e.g., Bonferroni correction) were made, which may lead to an increased likelihood of bias or random error. Consequently, the results, while offering valuable insights, should be considered preliminary and interpreted in consideration of these limitations. Additional research using larger, more diverse, and experimental designs will be necessary to validate and expand upon these findings.

## Conclusions

In this retrospective cross-sectional study of pediatric burns, the cultivated epidermal allograft was associated with clinically meaningful improvements over hydrocolloid dressings. Children treated with cultivated epidermal allograft demonstrated faster epithelialization, shorter hospital stays, and a markedly lower need for skin grafting. These advantages were noted in a cohort whose epidemiological characteristics correspond with global pediatric burn trends, primarily involving preschool-aged children with scald injuries and minimal TBSA involvement.

The results indicate that the cultivated epidermal allograft is a viable treatment for partial-thickness and indeterminate-depth pediatric burns. This is particularly important in settings with limited capacity. However, the retrospective design and single-center nature of the study restrict causal inferences. Despite these limitations, the magnitude and consistency of the differences observed support the consideration of cultivated epidermal allograft as a primary dressing strategy for appropriately selected pediatric burn wounds. To further confirm these benefits and establish optimal indications, timing, and cost-effectiveness across various care environments, prospective multicenter trials with standardized protocols and assessments of long-term scar quality and functional outcomes are necessary.
